# Alterations of Hair and Nail Content of Selected Trace Elements in Nonoccupationally Exposed Patients with Chronic Depression from Different Geographical Regions

**DOI:** 10.1155/2017/3178784

**Published:** 2017-03-12

**Authors:** Anna Błażewicz, Kuan-Yung Liao, Heng-Hsin Liao, Przemysław Niziński, Łukasz Komsta, Berislav Momčilović, Magdalena Jabłońska-Czapla, Rajmund Michalski, Andrzej Prystupa, Jarosław J. Sak, Ryszard Kocjan

**Affiliations:** ^1^Chair of Chemistry, Department of Analytical Chemistry, Medical University of Lublin, Chodzki 4a St., 20-093 Lublin, Poland; ^2^Homu Clinic, No. 61, Dongping Rd., East Dist., Tainan City 701, Taiwan; ^3^Chair and Department of Medicinal Chemistry, Medical University of Lublin, Jaczewskiego 4, 20-090 Lublin, Poland; ^4^Institute for Research and Development of the Sustainable Eco Systems, Srebrnjak 59, 10000 Zagreb, Croatia; ^5^Institute of Environmental Engineering of Polish Academy of Sciences, Sklodowska-Curie 34 Street, 41-819 Zabrze, Poland; ^6^Chair and Department of Internal Diseases, Medical University of Lublin, Staszica 16, 20-081 Lublin, Poland; ^7^Department of Ethics and Human Philosophy, Medical University of Lublin, Staszica 4/6, 20-059 Lublin, Poland; ^8^Department of Nephrology, Medical University of Lublin, Jaczewskiego 8, 20-954 Lublin, Poland

## Abstract

The aim of this study was to determine if altered levels of selected trace elements manifest themselves during chronic depression. To identify elements strongly associated with chronic depression, relationships between the elemental contents of hair and nails and the interelement correlations were checked. Inductively coupled plasma mass spectrometry and ion chromatography were used to evaluate the contents of Zn, Cu, Co, Pb, Mn, and Fe in hair and nail samples from a total of 415 subjects (295 patients and 120 healthy volunteers). The study included logistic regression models to predict the probability of chronic depression. To investigate possible intercorrelations among the studied elements, the scaled principal component analysis was used. The research has revealed differences in TE levels in the group of depressed men and women in comparison to the healthy subjects. Statistically significant differences in both hair and nails contents of several elements were observed. Our study also provides strong evidence that the intermediary metabolism of certain elements is age- and gender-dependent. Zn, Mn, Pb, and Fe contents in hair/nails seem to be strongly associated with chronic depression. We found no statistically significant residence-related differences in the contents of studied elements in nonoccupationally exposed patients and healthy subjects.

## 1. Introduction

Depression is a very common condition in the general population, and it has serious health implications. According to the World Health Organization (WHO), 350 million people worldwide are affected by depression [1] and major depressive episodes may be foreshadowed by periods of dysthymia (chronic, mild depression) [2]. Patients with depression report a variety of symptoms that interfere with their day-to-day lives. Individuals suffering from depression will commonly lose interest in the activities which they would normally enjoy. Depressed individuals also lose their appetite, become sleepless, often suffer low self-esteem, and have difficulty in concentrating. Serious clinical depression may lead to a social withdrawal and self-neglect [3]. The cumulative, repeated episodes of depression may also contribute to developing some sort of inflammatory process in the body [4]. Depression is regarded as a multifactorial disorder with many causes [5]. Genetic, neurological, hormonal, immunological, and neuroendocrinological disorders have all been implicated as important depression mechanisms. Gender and developmental factors can further alter these etiological factors [6].

Recently, numerous studies have provided valuable information regarding the involvement of essential chemical elements in psychiatric disorders [7, 8]. According to certain preclinical and clinical studies, such elements as zinc, magnesium, lithium, iron, calcium, and chromium are involved in the development of depression. It has been reported that supplementation of a low dose of an antidepressant with a specific chemical element can reduce unwanted side effects in different types of depression [9]. Although the exact mechanism of such an action is not fully understood, some chemical elements, such as magnesium, have long been successfully used as a supplement in the treatment of this condition [10]. The role of other chemical elements in depression is even less understood, although their importance in the physiology and pathology of the nervous system is indisputable [11]. Dysregulation of specific elements in the body often coincides with neurodegenerative and neuropsychiatric disorders. Frequently, this disruption in “normal” element content affects more than one element at a time, suggesting that the imbalance of one element must somehow affect that of others, upsetting the normal homeostasis [12]. Research findings indicate the warranted need of searching for trace elements (TE) biomarkers and mutual relationships between elements and etiology of a disease [13–15].

Analysis of TE in human tissues belongs to the challenges of today's analytical and clinical chemistry [16]. Such analysis is especially useful for quick detection of overall nutritional status and deficiency/toxicity studies. Hair and nail samples have been used frequently for the assessment of environmental and occupational metal exposure [17]. Nails provide an alternative sample medium. Both types of samples collected in a noninvasive way can serve as sources of valuable information about human metabolism. Validated procedures for the determination of elements in hair and nails are very scarce [18]. Due to the lack of standardised sample preparation procedures of hair/nail samples [19], there are still analytical problems that significantly impact the findings, which can lead to skewed results in publications and problems with comparisons between different studies [20]. Additionally, washing procedures used in hair elemental analysis can have significant effect on the internal elemental signal levels in hair [21].

Inductively coupled plasma mass spectrometry (ICP-MS) is the most versatile technique for determination of trace elements [22, 23]. Ion chromatography (IC) is an intensively developing technique for simultaneous determination of ions in samples of complex matrices [24].

The primary objective of this study was to evaluate the content of zinc, copper, manganese, iron, cobalt, and lead using optimised sample preparation method and two validated analytical methods (i.e., ICP-MS and IC). Both essential and toxic TE contents were evaluated in patients diagnosed with depressive disorder and compared with healthy subjects. We aimed to determine whether TE levels could be factors that significantly influence the risk for the development of depression in humans. An attempt was made to identify relationships between element content in hair and nails and interelement correlations to identify the elements and matrices that can be further used for the biological monitoring of depression. We also aimed to compare the content of selected trace elements in the samples taken from two populations coming from different geographical regions, that is, from Taiwan and from Poland.

Although many papers exist on toxic metals and microelements in human health and disease, studies on elements and patients suffering from persistent depressive disorder are sparse. Our study provides new data on toxic and trace elements that are associated with depressive disorders. We present here the studies replicated in two independent laboratories, what is more, with the use of two different analytical techniques, involving patients with the same disorder but coming from different geographical locations.

## 2. Materials and Methods

### 2.1. Patients and Samples

Our studies have been carried out in accordance with the Code of Ethics of the World Medical Association (Declaration of Helsinki) for experiments involving humans. The study protocol was reviewed and approved by the Ethics Committee of the Medical University of Lublin, Room 128, Al. Racławickie 1, 20-059 Lublin, Poland, and Homu Clinic, No. 61, Dongping Rd., East Dist., Tainan City 701, Taiwan (KE-0254/293/2013).

Written informed consent was obtained from all participants. The study description and questionnaires were translated into the native language of the participants. Doctors performing sample collection and physical examination also gave verbal instructions. The diagnosis was made following the DMS-4 criteria [3]. Patients were diagnosed with dysthymic disorder (chronic, mild depression), currently replaced by* persistent depressive disorder* [25]. All laboratory data were anonymous.

Patients underwent an extensive clinician interview and comprehensive laboratory work-up and completed a questionnaire detailing their personal and medical history. The information required included gender, age, BMI, place of residence, hair colour, personal habits, occupation, possible metal exposure, and use of medical treatments. The participants were not on any special diets. They could not be on antidepressive medication or neuroleptics, nor could they be taking any supplements for the three months before samples were collected. Other exclusion criteria included a history of smoking, current pregnancy, acute and chronic infections, autoimmune, allergic, neoplastic, and endocrine diseases and other significant physical ailments or injuries, including surgery within the last six months. Volunteers for the control group were selected from patients who were at the clinic for routine check-ups and were healthy with no lifetime history or current diagnosis of any psychiatric disorders. The controls underwent the same procedures and exclusion criteria as the studied group.

Trace elements (Mn, Co, Cu, Zn, Pb, and Fe) were determined in samples of human hair and fingernails collected between 2013 and 2014 from 215 inhabitants of southern Taiwan region and patients of Homu Clinic, Tainan City, Taiwan, and from 200 Polish inhabitants of Lublin region (south east region of Poland) who were enrolled in the study between 2014 and 2016. The entire analytical process was repeated twice.

Tests were performed in 415 adults who constituted the studied group (among 295 patients, 102 Taiwanese women and 53 men, age 33 to 73 y, the average age: 57.4 y, and 85 Polish women and 55 men, age 39 to 70 y, the average age: 59.2 y) and the control group, that is, 120 healthy volunteers (60 from Taiwan and 60 from Poland, among them 35 Taiwanese women and 25 men, age 29 to 71 y, the average age: 53.1 y, and 40 Polish women and 20 men, age 26 to 76 y, the average age: 49.3 y). Samples were collected by the use of stainless steel scissors. Hair was collected from the nape of the neck (less than 3 cm from the scalp) from patients who declared no occupational exposure to metals or metalloids. No samples were coloured or treated. The weight requested for each specimen was approximately 100 mg.

### 2.2. Sample Preparation Procedure

All samples of hair and nails underwent the washing procedure as follows: each sample was soaked in deionised water and later in acetone and again in deionised water with a resistivity of 18.0 MΩ cm (Millipore, Bedford, MA, USA). Each washing stage took 10 min. After drying in an oven (30 min at 100°C) they were weighed. To prevent possible loss of the sample, the weighing stage was after washing and drying. The samples collected from the participants of our study (approx. 100 mg) were divided (using stainless steel scissors) into 3 pieces to repeat the digestion procedure 3 times. The test portion mass was 30 mg. Next, samples were digested with 10 mL of digestion mixture, that is, 3 mL of 69.0% nitric acid (HNO_3_) solution + 7 mL of 30% m/m (H_2_O_2_). Both reagents were of Suprapur grade (Merck, Darmstadt, Germany). The digestion was carried out in NovaWAVE Microwave Tunnel Digestion System (SCP Science, Canada) using Teflon® vessels. The microwave-assisted sample preparation was conducted in a closed system. To improve the recovery of analytes, a careful optimisation of the digestion procedure was performed. To limit the consumption and number of reagents, the following parameters were taken into account: composition and volume of reagents, time of the procedure, and temperature. Regardless of sample type, the time of the digestion procedure did not exceed 30 min, including the cooling stage. The temperature was set at 180°C. The applied conditions minimise the possibility of sample contamination and loss of analytes during the entire procedure.

Deionised water with a resistivity of 18.0 MΩ cm was used for dilution (1 : 10 v/v) of the sample solutions after digestion.

### 2.3. Analytical Methods of TE Determination

Currently, ICP-MS is the most common method utilised for analysis because it enables accurate, precise, sensitive, and rapid multielement analysis of samples with complex matrices. Unfortunately, ICP-MS is not free from interferences during the determination of certain elements, for example, Fe in the presence of multiple other elements. Because the role of Fe is relevant in depression [9], our study was expanded to include its determination using ion chromatography (IC) [26]. The quantitative analyses of metals and metalloids were carried out with an ICP-MS spectrometer (Elan DRC-e 6100 model, Perkin-Elmer, USA). The spectrometer was optimised daily with a 10 *μ*g/L solution (Mg, Cu, Rh, Cd, In, Ba, Ce, Pb, and U) in 1% HNO_3_ (Elan 6100 Setup/Stab/Masscal produced by Perkin-Elmer). The spectrometer was optimised to provide maximal intensity for ^24^Mg, ^115^In, and ^238^U and minimal values for CeO/Ce (below 3%) and Ba^2+^/Ba (below 3%). All solutions of multielemental (Merck, Germany) and monoelemental (Merck, Germany) ICP-MS standards were prepared daily by the dissolution of the reference materials in water and used for the calibration. One point calibration was performed. Standards, blanks, and samples were measured with ^103^Rh as the internal standard (10 *μ*g/L, Merck, Germany). 10 *μ*g/L Rh solution was introduced into all solutions online, with the second tubing on the peristaltic pump.

HPIC analyses were performed on a Dionex DX-500 ion chromatograph (Dionex, Sunnyvale, CA, USA). Iron(III) cations were determined using an isocratic elution with 7.0 mmol of pyridine-2,6 dicarboxylic acid (PDCA), 66 mmol of potassium hydroxide, 5.6 mmol of potassium sulphate, and 74 mmol of formic acid as mobile phase, IonPac CS5A (250 mm × 4 mm ID, Dionex, Sunnyvale, USA) as the separation column and spectrophotometric detection (at 530 nm) after the postcolumn derivatisation with the use of 0.5 mmol of 4-(2-pyridylazo) resorcinol (PAR), 1.0 mol of 2-dimethylamino-ethanol, 0.3 mol of sodium bicarbonate, and 0.50 mol of ammonium hydroxide dissolved in deionised water. Appropriate concentrations of standards were prepared from 1 g/L stock standard solutions (Merck, Darmstadt, Germany). The operating parameters of IC and ICP-MS measurements are as follows: 

*HPIC*

  Stationary phase: ion exchange column IonPac CS5A (Dionex Co., Sunnyvale, USA), 2 × 250 mm
  Guard column: IonPac CG 5A (Dionex Co., Sunnyvale, USA), 2 × 100 mm
  Detection: absorbance, *λ* = 530 nm
  Eluent flow rate: 0.3 ml/min
  Postcolumn reagent flow rate [ml/min]: 0.15 ml/min
  Sample loop: 25 *µ*l
  Temperature: 25 ± 1°C
  Operating pressure: 1900 psi
  Number of replicates: 3

*ICP-MS*

  RF Power: 1125 W
  Plasma gas flow: 15 l/min
  Nebulizer gas flow: 0.76–0.79 l/min
  Auxiliary gas flow: 1.15 l/min
  Nebulizer: cross flow
  Plasma Torch: quartz
  Sample flow: 1 ml/min
  Scanning mode: peak hopping
  Dwell time: 100 ms
  Sweeps/reading: 20
  Number of replicates: 3

### 2.4. Validation Parameters

The detection limits were calculated as the average of six blank sample signals plus three times (and ten times for the limits of quantification) standard deviation of the signals obtained from blank samples. An acidified water sample was used to prepare calibration standards and dilution of all solutions and real samples. To assess the accuracy of the methods, the following certified reference materials were used: CRM NIES number 13 human hair and SRM 1643e-trace elements in water (National Institute of Standards and Technology (NIST) Gaithersburg, MD, USA).  Table 1 presents the fundamental validation parameters in the applied analytical methods of trace element determinations.

Statistical analysis was carried out using R 3.1., an open source software using built-in routines and “rpart” package [27].

## 3. Results and Discussion

The normality of results was checked by the Shapiro-Wilk normality test. Almost all variables exhibited significant (at 95% level, with almost all at 99.9% level) deviance from normality, except for Cu content in nails. The distribution of the results was significantly positively skewed, which was confirmed by D'Agostino test for skewness under the null hypothesis of normality.

The correlation of elements' content with age was done by the Pearson correlation coefficient with the appropriate test for significant correlation. No significant correlations were found between element contents and age. The Spearman coefficient with the test gave consistent results.

The univariate difference of TE levels between healthy volunteers and patients (for each element separately) was checked using the Wilcoxon test. Significant differences were found for the elements' content in hair, namely, Co (*p* = 0.017), Cu (*p* = 0.0016), Fe (*p* = 0.024), Mn (*p* = 2.7*e* − 05), and Zn (*p* = 2.8*e* − 08), and for content in nails, namely, Cu (*p* = 0.0072), Fe (*p* = 1.2*e* − 08), Mn (*p* = 1.4*e* − 05), Pb (*p* = 0.0020), and Zn (*p* = 2.4*e* − 06).

In an analogous manner, such differences between genders were tested. The content was found to be significantly different between genders for the elements Co (*p* = 0.0025), Cu (*p* = 0.00021), Zn (*p* = 0.042) in hair and Co (*p* = 3.1*e* − 06), Cu (*p* = 0.00095), and Fe (*p* = 0.032) in nails. To investigate the possible interactions between depression, gender, and age, the appropriate linear models were built. These models applied the content of each element as modelled variables, whereas age, gender, and depression were used as predictors. The possible interactions were as follows: gender-age (difference in slope, when there is another gender), gender-depression (cross-difference in content level), and depression-age (difference in slope between the control and studied groups). Despite confirming earlier conclusions found by the Wilcoxon test, the following interactions were found: (i) For Fe content in hair, a significantly different dependence of element content on age between genders was found (*p* = 0.016); the content slightly increased with age in women, whereas it decreased with age in men. (ii) Significantly lower levels of elements in healthy men (gender-disease interaction) were found for content in nails of Co (*p* = 0.018) and Cu (*p* = 0.0021). The opposite behavior (higher levels in healthy men) was found (but significant at 90% level only) for the content in nails of Zn (*p* = 0.030). (iii) Depression caused a significantly lower increase with age for Fe content in hair (*p* = 0.025).

The significant elevation in the concentration of manganese and significant decrease in the concentration of zinc were observed in the hair and nail samples of depressed patients.

After we had run an ANOVA test and found significant results, we performed Tukey's HSD to find out which specific groups' means (compared with each other) were different. The test compared all possible pairs of means, that is, for depressed women, depressed men, healthy women, and healthy men. In hair, we found that cobalt in female group with depression was significantly decreased when compared to control group and depressed men. The opposite result was found for copper in hair (significantly increased in depressed female group). For iron and lead in hair there were no significant differences among each group, whereas for manganese and zinc significantly different contents were found between both control groups and between studied groups. In nails, significant differences between both sexes (each female and male group) were identified for cobalt. For copper in nails significant differences were found between healthy men and other groups. Iron content in nails was significantly different between each healthy and each studied group (higher in the group with depression), content of manganese in nails was significantly higher in group with depression, content of lead in nails was significantly different between healthy men and depressed men, and zinc content in nails was significantly different in each group (the lowest content was found in depressed women).

**Figure 1:**
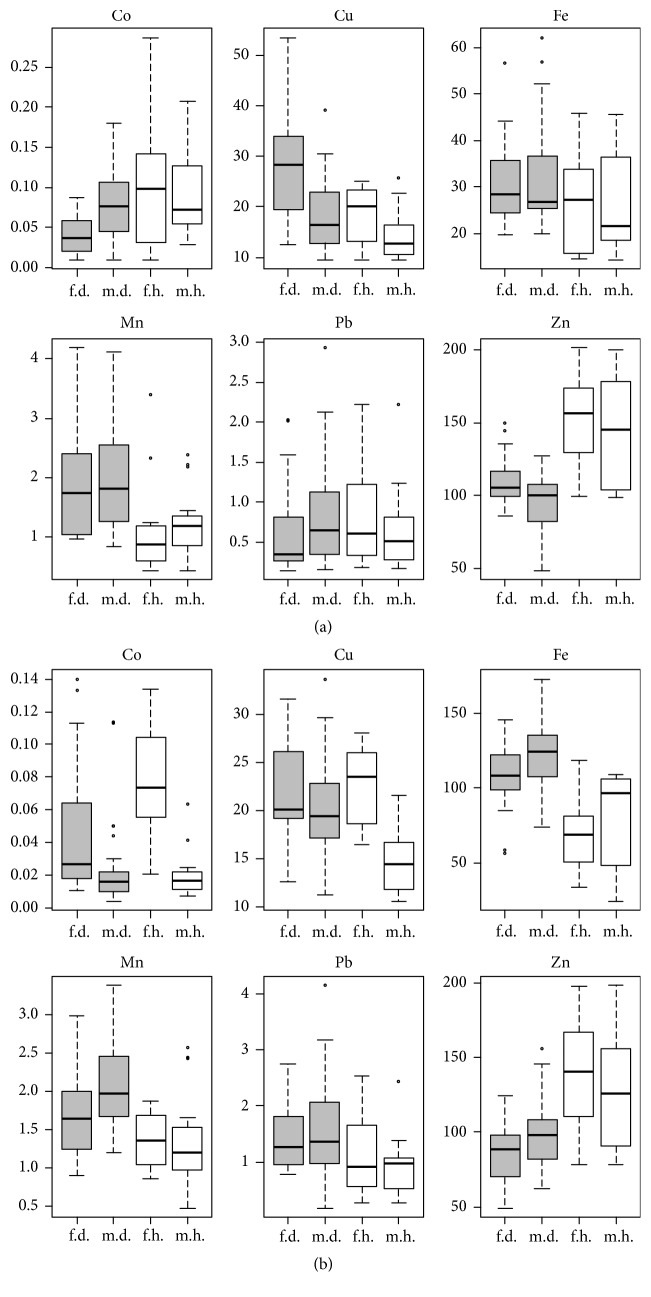
(a) Hair trace elements contents (mg/kg dry mass) in depressed (d) and healthy (h) women (f) and men (m). (b) Nail trace elements contents (mg/kg dry mass) in depressed (d) and healthy (h) women (f) and men (m).

Figures 1(a) and 1(b) present the contents of the elements organised according to gender and studied versus the control group. The contents are presented in mg/kg dry mass.

To investigate possible intercorrelations between elements, scaled principal component analysis was used. This data mining method treats all patients as points in multivariate space and tries to find an angle of a two-dimensional plane (as a so-called subspace) to represent the highest variance of the data. Projection onto this plane, understood as a “shadow” of the points, is the best possible representation of objects (patients) on a two-dimensional plot. However, the quality of the two-dimensional representation depends on structure of the data; that is, the more the intercorrelations, the better the ability to compress. Only 18% of total variance was explained by the two first principal components. This proves that the levels of elements are very independent and it is difficult to find any multivariate intercorrelations or visible trends in these data. The subjects had no tendency to be clustered. The lack of intercorrelations can be proven by investigation of loading vectors, that is, the projection of original axes onto the two-dimensional plane found by the analysis. In this case, loading vectors were randomly and uniformly distributed in all directions.

Next, the multivariate discriminant models (equation differentiating persons with and without depression) were created with scaled PLS-DA (Partial Least Squares-Discriminant Analysis) for hair, nails, and the total dataset. This method is analogous to the LDA (Linear Discriminant Analysis) but instead of ordinary least squares regression uses the PLS (Partial Least Squares) regression. Having a matrix, containing *n* rows (subjects) and *p* columns (elements) and a column vector *y* containing 1 value for depression and −1 for control, the method tries to find an estimator for predicting this *y* vector from *X*. This estimator is then used for further prediction, and negative *y* means healthy, whereas positive indicates depression. In the case of multidimensional and intercorrelated data, it is the natural choice to use PLS regression instead of the classical one.

**Figure 2:**
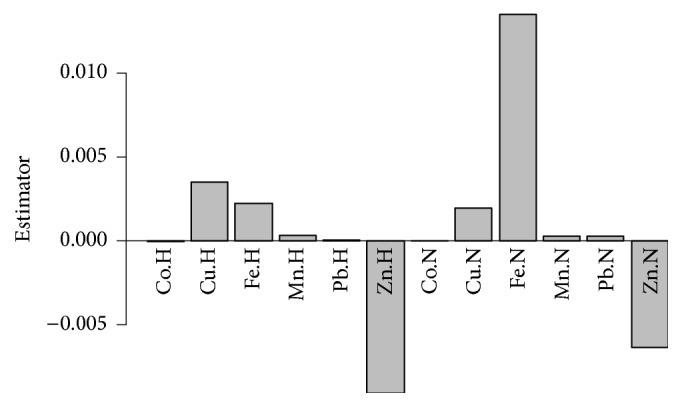
The values of PLS-DA estimator, discriminating subjects with disorder from control group, made with the whole dataset (H-hair, N-nails).

The complexity of two PLS factors was chosen with cross-validation in all cases. In the case of hair, seven healthy persons were misclassified as sick and two persons with depression were misclassified as healthy. In the case of nails, five healthy persons were misclassified as sick and two persons with depression were misclassified as healthy, respectively. A total model resulted in the misclassification of two and one persons, respectively. The estimators of PLS-DA model are shown in Figure 2. Positive values mean an increase of the element content during depression, whereas negative means a decrease. It should be noted that the estimator values are scaled, so two similar values do not mean similar impact of the element, but similar impact scaled to its variance.

To obtain a formula for depression probability prediction, a logistic regression was used with stepwise variable selection against the Akaike information criterion (AIC). Three separate models were built and found: for hair, nails, and the augmented dataset.  Table 2 presents the most significant variables (elements).

All of the methods detected similar variables, and this finding additionally confirms that these elements may, in fact, be relevant for chronic depression. It should be noted that the results do not predict the severity of depression.

From our study, it is clear that there is a statistically significant difference in TE content of hair and nails between the depressed patients and the healthy subjects. It is important to take into consideration that all the participants were not on any special diets. Obviously the regular diet was different in the two countries, but the trend in altered trace elements profile was significant for dysthymic patients, regardless of their place of residence. It may suggest that not only nutritional factors are responsible for altered levels of investigated elements in hair and nails of dysthymic patients. Our findings may indicate a possible relationship between chronic depression and deficiency or excess of some TE as well as alterations in their metabolism.

The lack of strong correlation between TE contents and place of residence of our participants and similar trend in alteration of TE content in chronic depression indicate that trace analysis may serve as a useful tool for the purposes of prevention (Table 3).

Zinc deficiency can lead to numerous complications such as stunted growth, diarrhoea, impotence, hair loss, eye and skin lesions, impaired appetite, and depressed immunity. Some cross-sectional studies associate a low Zn intake with depression in women [28, 29], whereas a 20-year prospective follow-up study suggests that dietary Zn intake was not associated with an increased risk of depression in men [30]. Recent published findings [31] suggest such an association with a greater incidence of depression in both men and women. Very recently, Lehto et al. [32] have emphasised that Zn probably will not be the golden remedy for depression due to a high heterogeneity in the etiopathogenesis of depression. Reported clinical data indicate decreased serum Zn levels in human depression. Altered Zn levels could be a potential marker of depression [33–36]. Greater depression severity was associated with a relatively greater Zn deficiency [37]. Some studies suggested that low Zn levels could be related to the activation of cell-mediated immunity in depression [38]. Studies performed by Siwek et al. [39] suggest three possible reasons why lower Zn levels were observed in depression (i.e., nutritional deficiency, hyperstimulation of the hypothalamic-pituitary-adrenal axis and/or inflammatory/acute phase response, and oxidative stress). The authors stressed that serum Zn would not make a good specific marker of depression because it rather serves better as a marker of immune activation and oxidative stress. Ghanem et al. [40] found a significantly lower level of Zn in hair samples of depressed patients in comparison with healthy subjects, which is consistent with our results. According to our knowledge, there are no published studies of the Zn content in the nails of depressed patients. It is interesting that the difference in the decrease of Zn between nails and hair in both groups is so similar.

Certain studies imply that relatively low amounts of Mn exposure could affect mood because even low levels of exposure result in neuropsychological effects [41]. The issue of Mn's role in psychiatric and neurological diseases is extremely important, because even after exposure has ceased, and serum, hair, and urine levels return to normal, the progress of neuropsychiatric symptoms can continue. It is suggested that Mn^2+^ and Mn^3+^ can react with dopamine to create reactive oxygen species, which is known to cause damage to the dopaminergic neurons [42]. The exact mechanism of Mn and dopamine interaction-induced cell death remains unclear [43]. It is known that disturbances in Fe homeostasis can contribute to Mn toxicity [44]. Fe is required for normal cellular function and structure, playing roles in DNA and neurotransmitter synthesis [45]. It was suggested that even in nonanaemic patients abnormal Fe levels may alter mood and behavior in a similar way to depressed subjects. Severe iron deficiency and resulting anaemia are associated with impaired cognitive functions and abnormal neuropsychological development [46]. According to Młyniec et al. [9] depression is a multifactorial disorder and Fe may have a positive or negative effect on depression. Psychological stress has been shown to cause Fe deposits in the brain. One study showed reduced release of Fe during inflammation and suggested that lower Fe levels may be a result of depression [47]. Our studies show that depressed patients can be expected to have an increase of Fe content in hair and nails even when no inflammatory state is present. Unlike with the proportional decrease of Zn in both matrices, the observed increase of Fe was not proportional between the hair and nail samples.

The mechanisms of Pb neurotoxicity and the effects of Pb on neuronal death, on intraneuronal regulatory mechanisms, on neurotransmission, on transmitter release, and on transport of thyroid hormone have been studied extensively [48]. Indirect neurotoxic effects of Pb include anaemia as a result of interference with heme synthesis and through decreasing Fe absorption from the gut [49].

As for the other elements that were quantified in our study, although no strong associations were found between their content and the prevalence of depression in the patients studied, it should be noted that our results found an increase of Cu in both types of samples. This observation is consistent with data of studies undertaken by Ghanem et al. [40]. Cu is involved in the absorption, storage, and metabolism of Fe; therefore higher Cu levels could be responsible for the higher levels of Fe in depression. Cu is required for dopamine synthesis, and dopamine in the brain plays an important role in depression [50].

The observed associations could provide insight into the etiology of depression, helping generate new hypotheses for further research and perhaps contribute to future healthcare planning. We realise that there is a need of a speciation analysis in relation to certain elements determined in biological matrices because various chemical species can indicate different biological effects. Unfortunately, some sample pretreatment procedures interfere with speciation studies, and therefore the final determination refers to “total” TE concentration in hair or nail samples. The ideal analytical strategy in which all species of all elements are determined simultaneously followed by the same pretreatment procedures still does not exist [20].

Our studies have important strengths, for example, application of the most suitable analytical techniques taking into account the complexity of samples' matrices. These techniques were evaluated with the appropriate reference materials to ensure high quality of the entire analysis. Moreover, in our study we used two analytical techniques which allowed us to perform a multielement determination, including Fe, which is usually underestimated in ICP-MS studies.

The determination of Fe by ICP-MS is not recommended, especially in samples with complex matrices because of polyatomic interferences from different isotopes produced by O, Ar, and Ca on ^56^Fe (^40^Ar^16^O^+^ and ^40^Ca^16^O^+^) and ^57^Fe (^40^Ca^16^O^1^H^+^, ^40^Ar^16^O^1^H^+^). The interferences caused by ^40^Ar^16^O^+^ and ^40^Ar^16^O^1^H^+^ on ^56^Fe and ^57^Fe, respectively, may be reduced by using a shield torch, which unfortunately can produce other polyatomic interferences [51]. The next advantages that are not negligible include ease of collection and excellent stability of the samples.

We present the associations between hair and nail TE status after age and gender adjustments, and the exclusion criteria of our study limit additional factors (variables) that may affect the content of TE. Patients with other psychiatric pathologies were excluded in our study. Neither the participants nor the persons conducting the experiment had access to key information that could interfere with or influence the results.

There are different methods of analysis currently in use with different procedures used to collect and prepare samples. Widely divergent reference ranges are reported for the normal individual depending on which method of analysis is used, making it difficult to compare the results between different studies. External contaminants pose a significant concern and the proper pretreatment of samples should be used to limit such possibilities. There are a variety of factors that affect the content of TE in hair/nails in and of itself, namely, age, gender, diet, hair type, location of hair, and so forth. Despite these limitations, TE analysis of hair and nails remains an area of continuing interest and active research. However, the analytical data obtained must be interpreted with caution and forethought.

## 4. Conclusions

Despite a growing number of studies undertaken in the field of TE determination in various disorders, the role of TE in depression has not been thoroughly elucidated. A reliable analysis of easily accessed samples of hair/nail could provide an answer to the still unsolved question of whether deficiency of certain elements or excess toxicity of other elements manifests itself during depression. Mutual interactions between elements in this disorder should also be taken into consideration for useful diagnostic information.

The results of this study indicate various significant dependence between TE levels, the sample type, age, and gender. Zn, Mn, Pb, and Fe contents in hair/nails seem to be strongly associated with chronic depression. Further cohort studies could be useful in definitely elucidating whether TE are good predictors for the development of depression or whether depression's metabolic influences are the cause of changes in TE levels. The significant elevation in the concentration of manganese and significant decrease in the concentration of zinc were observed in the hair and nail samples of depressed patients. For Fe content in hair, a significantly different dependence of element content on age between genders was found (*p* = 0.016); the content slightly increased with age in women, whereas it decreased with age in men. Significantly lower levels of elements in healthy men were found for content in nails of Co (*p* = 0.018) and Cu (*p* = 0.0021). The opposite behavior (higher levels in healthy men) was found (but significant at 90% level only) for the content in nails of Zn (*p* = 0.030). Depression caused a significantly lower increase with age for Fe content in hair (*p* = 0.025). The content of TE was found to be significantly different between genders for the elements Co (*p* = 0.0025), Cu (*p* = 0.00021), and Zn (*p* = 0.042) in hair and Co (*p* = 3.1*e* − 06), Cu (*p* = 0.00095), and Fe (*p* = 0.032) in nails. We found no statistically significant residence-related differences in the contents of Zn, Cu, Co, Mn, Fe, and Pb in nonoccupationally exposed patients with chronic depression and healthy subjects.

## Figures and Tables

**Table 1:** Validation parameters in applied analytical methods of trace elements determinations.

Analyte	Isotope	Operating range [*µ*g/L]	*V*_*R*_ [%]	*V*_*r*_ [%]	LOD [*µ*g/l]	LOQ [*µ*g/l]	*U* [%]	Recovery NIST 1643-e [%]
Mn	55	0.20–100	10.4	6.3	0.033	0.198	11.1	108.1
Co	59	0.01–100	4.6	4.6	0.002	0.012	10.0	109.6
Cu	65	0.38–100	5.8	3.5	0.064	0.384	12.4	96.1
Zn	66	2.50–100	11.4	7.6	0.181	1.086	14.6	80.3
Pb	208	0.21–100	13.9	11.3	0.036	0.216	18.0	105.7
Fe^*∗*^	—	0.30–5000	5.1	3.3	0.09	0.30	13.2	CRM NIES number 13 human hair 98.4

^*∗*^Fe^3+^ determined by IC; LOD: limit of detection; LOQ: limit of quantification. *V*_*R*_: relative standard deviation of reproducibility. *V*_*r*_: relative standard deviation of repeatability. nd: no data in CRM certificate. *U*: expanded uncertainty.

**Table 2:** The results of logistic regression (estimator value and its significance)^*∗*^.

	Estimate	Std. error	*z* value	Pr(>|*z*|)
Hair model				
(Intercept)	6.5	2.58	2.52	0.0119
Zn.H	−0.088	0.0274	−3.21	0.00131
Cu.H	0.204	0.0884	2.3	0.0213
Mn.H	1.71	0.692	2.47	0.0135
Co.H	−19.7	8.71	−2.26	0.0235

Nail model				
(Intercept)	−6.65	3.52	−1.89	0.0591
Fe.N	0.112	0.0368	3.03	0.00247
Zn.N	−0.101	0.0338	−3	0.00274
Cu.N	0.38	0.141	2.69	0.00719

Total model				
(Intercept)	−3.5	4.43	−0.791	0.429
Zn.H	−0.122	0.0408	−2.98	0.00286
Fe.N	0.0909	0.0293	3.1	0.00193
Cu.H	0.361	0.124	2.9	0.00373
Pb.N	2.7	1.08	2.51	0.012

^*∗*^The modelled equation computes depression probability from the most significant elements determined in hair and nails.

**Table 3:** Contents of studied elements (in mg/kg on dry mass basis) in hair and nails of studied and control groups according to the place of residence (*N* = 415).

Element	Sample matrix	Patients (*N* = 295) from		Mann–Whitney test	Healthy volunteers (*N* = 120) from		Mann–Whitney test
		Taiwan (155)	Poland (140)		Taiwan (60)	Poland (60)	
		Mean ± SD^*∗*^			Mean ± SD		
Zn	Hair	100.21 ± 31.56	99.16 ± 28.16	NS^*∗∗*^	147.85 ± 38.22	150.11 ± 35.44	NS
	Nails	92.85 ± 29.22	90.11 ± 24.62		131.8 ± 30.57	129.8 ± 28.14	
Cu	Hair	22.05 ± 11.14	23.18 ± 9.98	NS	15.81 ± 6.68	16.81 ± 10.41	NS
	Nails	20.59 ± 10.23	22.13 ± 11.33		17.93 ± 10.89	18.23 ± 9.91	
Mn	Hair	1.92 ± 0.55	2.42 ± 0.48	NS	1.19 ± 0.42	1.22 ± 0.39	NS
	Nails	1.93 ± 0.62	1.69 ± 0.54		1.35 ± 0.63	1.39 ± 0.93	
Co	Hair	0.06 ± 0.02	0.05 ± 0.01	NS	0.09 ± 0.03	0.08 ± 0.04	NS
	Nails	0.03 ± 0.01	0.02 ± 0.01		0.04 ± 0.01	0.05 ± 0.02	
Fe	Hair	31.3 ± 9.23	33.7 ± 10.22	NS	26.60 ± 5.48	24.90 ± 4.22	NS
	Nails	115.9 ± 51.21	118.4 ± 41.11		75.32 ± 20.26	78.00 ± 18.16	
Pb	Hair	0.74 ± 0.28	0.82 ± 0.33	NS	0.75 ± 0.32	0.73 ± 0.45	NS
	Nails	1.48 ± 0.75	1.52 ± 0.84		1.00 ± 0.49	0.099 ± 0.36	

^*∗*^SD: standard deviation; ^*∗∗*^NS: nonsignificant difference.
